# Positive Insights on Student Research Engagement in Academic Undergraduate General Practice: A Mixed Methods Study

**DOI:** 10.1111/tct.70409

**Published:** 2026-04-05

**Authors:** Nandakumar Ravichandran, Crea Carberry, Nia Clendennen, John Broughan, Geoff McCombe, Walter Cullen

**Affiliations:** ^1^ School of Medicine University College Dublin Dublin Ireland; ^2^ Clinical Research Centre, School of Medicine University College Dublin Dublin Ireland

**Keywords:** general practice, medical education, primary care, research engagement, summer programme

## Abstract

**Background:**

Research engagement during medical school enhances students' critical thinking, research skills, and competitiveness for future careers. The University College Dublin (UCD) Student Summer Research Awards (SSRA) programme provides undergraduate medical students with an opportunity to undertake supervised research projects. This study aimed to evaluate the feasibility and acceptability of the SSRA General Practice programme among medical students engaged in primary care research.

**Methods:**

A mixed‐methods study was conducted, including a secondary analysis of programme participation data (2016–2024) and a self‐administered survey distributed to former SSRA participants. Quantitative data were analysed using IBM SPSS Version 27, whereas qualitative responses were examined through thematic analysis.

**Results:**

Of 82 applicants, 45 students (54.8%) were selected for the SSRA General Practice programme, with 44 (97.7%) successfully completing it. Research dissemination was high, with 40 (90.9%) participants presenting at SSRA Poster Night and having their abstracts published. Additionally, 16 (36.4%) published in scientific journals, and 29 (65.9%) presented at national and international conferences. Among 18 survey respondents, the majority reported increased research skills (77.8%), satisfaction with supervision (77.8%), enhanced motivation for research (50.0%) and general practice careers (55.6%).

**Conclusion:**

The GP SSRA programme has demonstrated positive outcomes by increasing student engagement in research and reported greater interest in primary care research and general practice. Future efforts should focus on increasing funding, supervisor involvement and integrating structured research training into the medical curriculum.

## 1 Introduction

Research plays a pivotal role in medicine, especially in general practice (GP), where it significantly contributes to the advancement of medical knowledge, improves patient care and has a broader impact on socioeconomic development [1]. Encouraging medical students to engage in research is a widely supported initiative, but there is limited evidence on the most effective strategies for involving students in research activities [2]. The ‘not by chance, by choice’ publication by the Medical Schools Council England suggests that universities should collaborate to raise the profile of academic GPs by ensuring all students are encouraged to participate in scholarly activities, which should be visibly supervised by primary care leads [3].

Short‐term research training programmes provide valuable exposure to research for medical students. The first and second years of medical school offer the longest uninterrupted periods for research involvement [4]. Early engagement in research bridges the gap between information availability and student understanding, allowing for a deeper comprehension of the subject matter [5]. These programmes integrate clinical practice with academic inquiry, fostering critical evaluation, intellectual curiosity and essential skills in evidence‐based medicine. Additionally, early research experience enhances students' competitiveness for residency programmes, career advancement and networking opportunities [6].

The UCD General Practice Student Summer Research Awards (GP SSRA) programme is an undergraduate research initiative to support and showcase undergraduate research affiliated with the UCD School of Medicine. GP SSRA is a five credit, 8‐week elective module, providing medical students with the opportunity to undertake a supervised research project in primary care. This programme is open to students from any year of medical school. The programme allows the students to deepen their knowledge and understanding of primary care‐related issues while also providing the opportunity to publish a peer‐reviewed article [7]. This study aimed to investigate the feasibility and acceptability of the UCD GP SSRA programme from the perspective of the students involved in primary care research.

## 2 Methods

### 2.1 Study Design

To assess the feasibility and acceptability of the GP SSRA programme, a mixed methods approach was used. Proctor's implementation outcomes (acceptability, feasibility and sustainability) and the RE‐AIM framework (Reach, Effectiveness, Adoption, Implementation and Maintenance) were adopted to guide the study, ensuring systematic evaluation of implementation, reach and participant experiences [8, 9]. This included (a) secondary analysis of existing data, drawn from the research team's records on student engagement in the program, and (b) a self‐administered survey aimed at capturing students' views and experiences of the programme.

### 2.2 Recruitment, Setting and Participants

A self‐administered questionnaire was designed in November 2024, adapted from previously published Kirkpatrick‐based instruments used in undergraduate medical education research [10] (see Appendix A). Face validity was assessed by three academic GPs (CC, NC and WC) and three medical education researchers (NR, JB and GM). Because of the small sample size, internal consistency testing was not statistically meaningful; however, items were derived from previously validated Kirkpatrick‐aligned instruments.

Students who participated in the GP SSRA programme between 2016 and 2024 were invited to take part in the study (*n* = 38/45), excluding five whose contact details were unavailable, and one invitation was returned undelivered. Invitations were sent via email to complete the questionnaire. Along with the invitation, participants received an information leaflet, a consent form (which was integrated into the Google Forms survey), and the contact details of the lead investigator. Students were required to provide informed consent prior to participation in the survey. The survey was administered online through Google Forms, and responses were collected anonymously between 15 January 2025 and 9 February 2025.

#### 2.2.1 Inclusion Criteria

- Participants aged 18 years or older.
- Able to provide informed consent.
- Have participated in the GP SSRA programme.

### 2.3 Study Instruments

Secondary data on student engagement with the programme during the period 2016–2024 were collected from the research team's records. Students' self‐reported responses to questions on the effectiveness, acceptability and feasibility of the SSRA programme were collected through the self‐administered questionnaire. Open‐ended questions were also included to capture additional feedback on the acceptability of the programme and areas for improvement.

### 2.4 Data Analysis

Statistical analysis was performed using IBM SPSS Version 27.0. Continuous data are presented as median and interquartile ranges (IQR) or means SD where appropriate. Categorical data are presented as frequencies (percentages). The open‐ended responses of the survey were analysed by authors NR and JB and audited by the author GM. NVivo v12 software was used to perform data analysis identifying quotes for the relevant feasibility themes and developing the coding scheme by which data were categorized into their respective themes. All members of the research team maintained reflexivity throughout data analysis by having regular meetings in which identified themes were discussed and, when necessary, revised to reflect the various components of the model. Acceptability and feasibility of the programme were assessed using the reflexive thematic analysis approach designed by Braun and Clark [11] framework. The reflexive thematic analysis enabled us to use a data‐driven/bottom‐up approach in analysing the surveys to find themes that arise from the open‐ended responses provided by study participants.

### 2.5 Ethics

Ethical approval for the study was obtained from UCD Human Research Ethics Committee (LS‐C‐24‐314‐Cullen). Participants were fully informed about all study procedures through a participation information leaflet, and consent was obtained prior to their involvement in the study.

## 3 Results

### 3.1 GP SSRA Programme Engagement (Secondary Data) (2016–2024)

Between 2016 and 2024, 82 students applied for the GP SSRA programme. Of these, 45 students (54.8%) were selected based on their statements of research purpose and interest in primary care research. Among the selected participants, 44 students (97.7%) successfully completed the programme. Of those who completed the programme, 40 participants (90.9%) presented their research at SSRA poster night and had their abstracts published in both the SSRA Book of Abstracts and the Irish Journal of Medical Science. Additionally, 16 participants (36.4%) had their research published in scientific journals, whereas 29 participants (65.9%) presented their work at various national and international conferences, namely, the Association of University Departments of General Practice in Ireland (20 participants, 68.9%), the Society for Academic Primary Care (6 participants, 20.7%) and the European General Practice Research Network (3 participants, 10.3%) (Table 1). Additionally, 12 (27.3%) received funding. Of these, 8 (66.7%) were awarded the UCD‐based scholarship Mary J. Farrell Scholarship, whereas 4 (33.3%) received funding from Medisec Ireland [12, 13].

**TABLE 1:** Programme engagement data.

	*n* (%)
Number of participants who applied for the programme	82
Number of participants selected for the programme	45 (54.8)
Number of participants who completed the programme	44 (97.7)
Number of students funded	12/44 (27.3)
Number of students who presented at SSRA poster night	40/44 (90.9)
Number of students whose work was published in scientific journals	16/44 (36.4)
Number of students whose abstracts were published in the SSRA Book of Abstracts and in the Irish Journal of Medical Science	40/44 (90.9)
Number of students presented their work at national and international conferences	29/44 (65.9)

**FIGURE 1:**
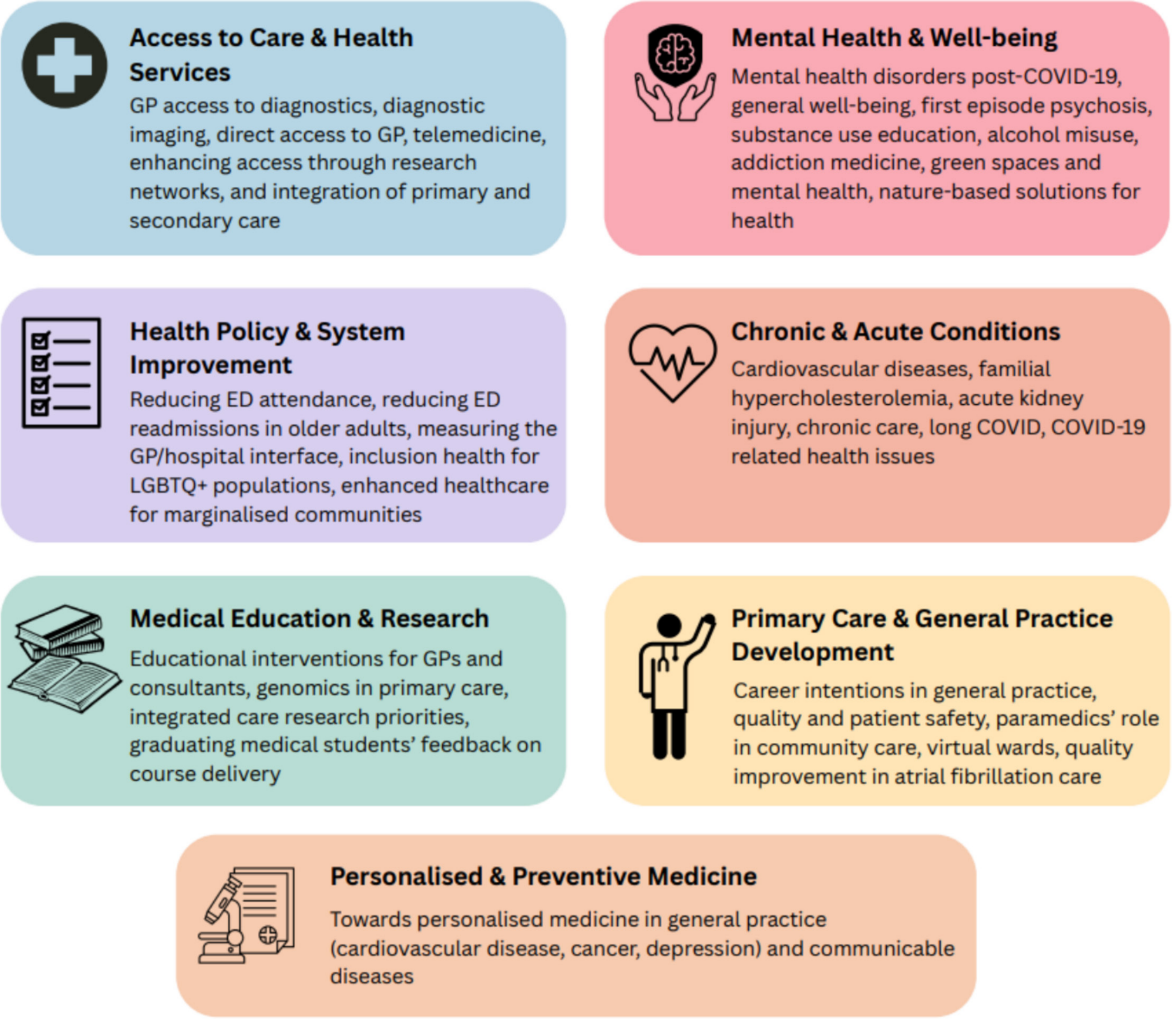
Topics studied in the GP SSRA programme 2016–2024.

The topics studied in the GP SSRA programme spanned various primary care and GP issues, including access to care, mental health, chronic and acute conditions, health policy, medical education and personalized medicine (see Figure 1).

### 3.2 Self‐Administered Questionnaire

**FIGURE 2:**
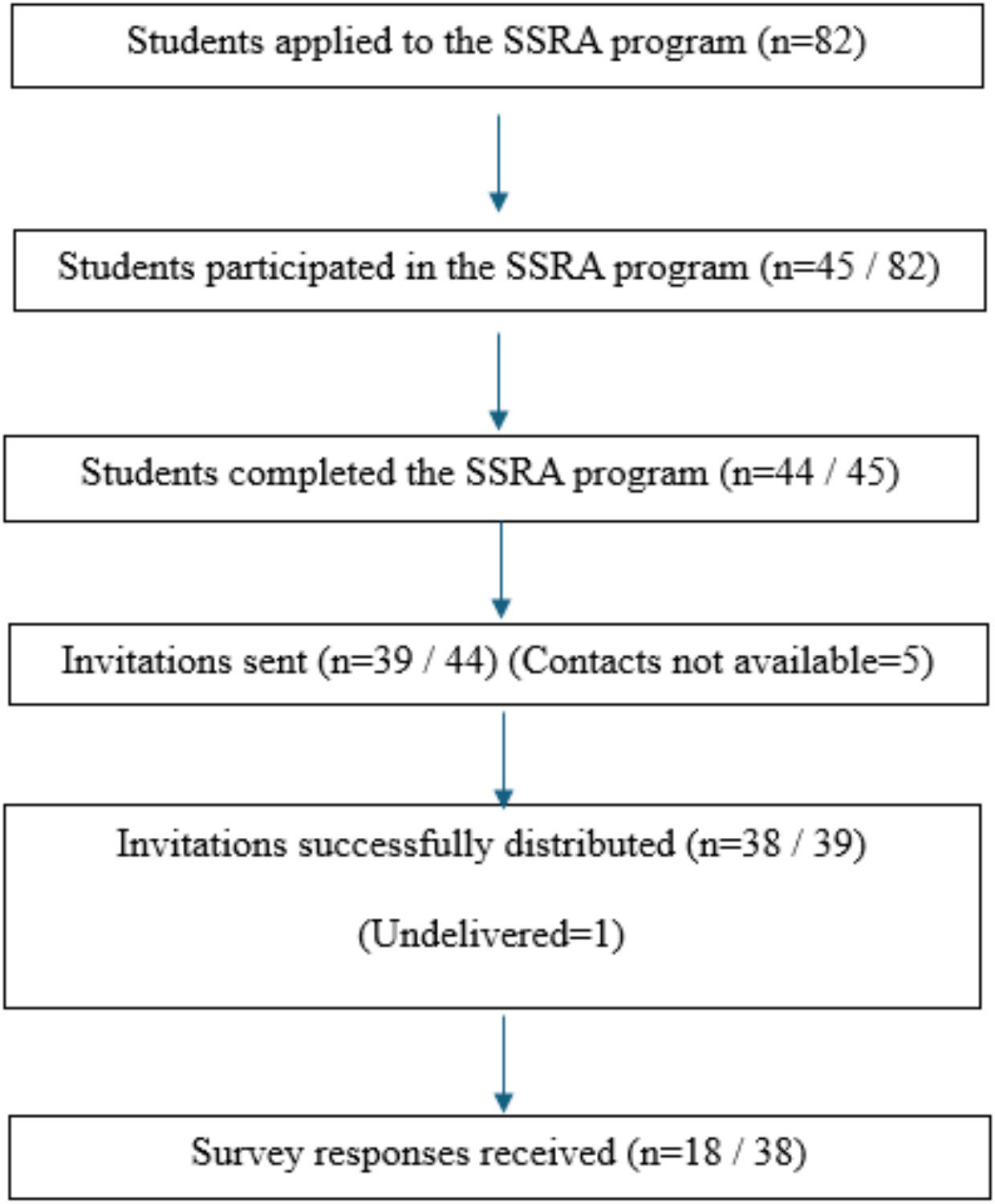
Distribution of self‐administered questionnaire.

Of the 44 participants who completed the GP SSRA programme, invitations to participate in the study were sent to 39 (88.6%) participants, excluding five whose contact details were unavailable. One invitation was returned undelivered, leaving 38 successfully distributed questionnaires. A total of 18 responses (47.3%) were received and included in the final analysis. Some nonresponses were likely due to participants having graduated and no longer being reachable through their university email addresses (Figure 2).

The mean age of the survey respondents was 23.9 years. Of the 18 respondents, 13 (72.2%) were female, and five (27.8%) were male. Twelve (66.7%) were students in their prefinal or residential year, and the remaining six (33.3%) were practising in various specialties, including plastic surgery, endocrinology, microbiology, obstetrics and gynaecology (OBGYN) and GP.

#### 3.2.1 Satisfaction of the Participants With the GP SSRA Programme

All the 18 respondents (100.0%) agreed or strongly agreed that they were satisfied with the programme. A majority (*n* = 14, 77.8%) strongly agreed that the programme enhanced their research skills. Furthermore, 50.0% (*n* = 9) strongly agreed that the programme increased their motivation to pursue a research career. Whereas 55.6% (*n* = 10) agreed it increased their motivation to pursue a career in GP, four students (22.2%) disagreed with the statement. Additionally, more than two thirds of the respondents (*n* = 14, 77.7%) strongly agreed or agreed that the programme expanded their professional network (Table 2).

**TABLE 2:** Participants' satisfaction with the GP SSRA programme (*n* = 18).

Criteria	Strongly agree	Agree	Neutral	Disagree	Strongly disagree
Satisfied with the programme in GP	7 (38.9)	11 (61.1)	0 (0.0)	0 (0.0)	0 (0.0)
My project was interesting	6 (33.3)	8 (44.4)	4 (22.2)	0 (0.0)	0 (0.0)
Improved research skills	14 (77.8)	4 (22.2)	0 (0.0)	0 (0.0)	0 (0.0)
Increased motivation to pursue a career in research	9 (50.0)	5 (27.8)	3 (16.7)	1 (5.6)	0 (0.0)
Increased motivation to pursue a career in GP	3 (16.7)	10 (55.6)	1 (5.6)	4 (22.2)	0 (0.0)
Expanded professional network	8 (44.4)	6 (33.3)	3 (16.7)	1 (5.6)	0 (0.0)
Supervisors were helpful	14 (77.8)	4 (22.2)	0 (0.0)	0 (0.0)	0 (0.0)

Of the 18 participants, 14 (77.8%) preferred individual meetings with supervisors, 8 (44.4%) preferred group sessions and 4 (22.2%) preferred webinars. Of the 18 participants, 3 (16.7%) reported group sessions as least preferred, 6 (33.3%) reported webinars as least preferred and 10 (55.6%) reported none. Most participants (15, 83.4%) agreed or strongly agreed that they liked the remote participation through Zoom or similar platforms.

#### 3.2.2 Qualitative Analysis of the Survey (*n* = 13)

Reflexive thematic analysis using the Braun and Clarke framework identified three key themes: attitudes towards research and GP, competencies and skills, and programme feasibility and acceptability [11].

**a. Attitude Towards Research and GP**

Participants described a clear shift in how they understood both research and GP as a result of their participation in the SSRA programme. Rather than viewing research as peripheral to clinical work, many came to see it as integral to professional development and to primary care practice. One participant reflected that ‘the program has given me a positive attitude towards research and increased my confidence in writing papers … I have now published several first author papers’ (P1), indicating both increased self‐efficacy and sustained scholarly engagement. Others described how the programme reshaped their perception of GP itself as a legitimate academic field, with one noting that it ‘introduced the idea of academics in general practice which was something I had not previously considered’ (P3). For some, this exposure generated new research ambitions within primary care, as illustrated by a participant who stated, ‘I will be considering pursuing further research in General Practice settings … as my project has brought up a number of interesting questions’ (P2). Together, these accounts show how the programme influenced both research identity and career‐related thinking about GP.

**b. Competencies and Skills**

Participants consistently highlighted the development of transferable research and communication skills that they perceived as valuable across clinical contexts. This included literature searching, critical appraisal, academic writing and the use of reference management tools. One participant emphasized the broad relevance of these competencies, stating that ‘these skills are universal research skills which are beneficial to pursuing any clinical role irrespective of the specialty’ (P3). Others linked these skills more directly to future patient care and teamwork, with one noting that ‘the research skills I developed … will help me stay updated with the latest, reliable and trusted approaches and the communication skills … will help my engagement with patients and my colleagues’ (P4). These accounts illustrate how participants understood research training not simply as an academic exercise, but as a foundation for evidence‐based and reflective clinical practice.

**c. Feasibility and Acceptability**

The programme was widely perceived as both feasible and acceptable, particularly because of its flexible structure, strong supervision and remote accessibility. Participants valued the balance between autonomy and support, with one describing it as ‘a great introduction to the world of research … very flexible and allowed me to balance my time well’ (P5). Access to funding and mentorship further enhanced engagement, as another participant explained that ‘receiving support and funding … afforded an opportunity for me to further my understanding of Primary Care services’ (P2). Many participants also highlighted the benefits of a hybrid model, especially for those not based in Dublin, with one noting that ‘a few in‐person workshops, meetings etc. will be positive for building co‐operation and communication among students and students with faculty. However, the remote aspect of this program allows student to conduct research from home if you don't live in Dublin or away during the summer which makes this very accessible’ (P6). These perspectives demonstrate how structural features of the programme supported participation, equity and sustained engagement.

## 4 Discussion

This study provides a multiyear evaluation of a GP‐based undergraduate summer research programme, demonstrating how structured, mentored research opportunities embedded in primary care can support research capability development, scholarly engagement and evolving professional identities among medical students. This aligns with studies from Australia, the United States and the United Kingdom, where research programmes in GP have significantly increased scholarly output and helped students develop academic identities within the GP setting, alongside increased scientific scepticism [14, 15, 16]. Rather than reiterating programme outputs, this discussion situates the findings within the broader literature on undergraduate research training and considers their implications for clinical education, implementation and transferability across settings.

A key contribution of this work lies in its focus on GP as a research environment, an area that remains underrepresented in evaluations of undergraduate research initiatives. Whereas prior studies of undergraduate summer research programmes have predominantly focused on biomedical and hospital‐based settings, our findings extend this literature by demonstrating that similarly strong educational and professional development outcomes can be achieved within a GP research environment [1, 17, 18]. The GP SSRA programme demonstrates that primary care offers a fertile context for student research engagement, encompassing health services research, policy‐relevant inquiry, education research and patient‐centred outcomes. This aligns with international calls to strengthen research capacity within primary care as a cornerstone of sustainable health systems.

Participants' accounts suggest that early exposure to GP‐based research can broaden perceptions of academic career pathways and challenge assumptions that research is confined to tertiary or laboratory settings. These findings echo prior work showing that authentic, mentored research experiences contribute to professional identity formation and sustained engagement with scholarship, regardless of eventual specialty choice [19, 20, 21]. Importantly, the competencies described by participants, critical appraisal, academic writing, literature synthesis and reflective thinking are transferable skills valued across clinical and interprofessional roles, enhancing the relevance of this model beyond medicine alone.

Mentorship emerged as a central mechanism underpinning programme impact. Consistent with evidence highlighting the role of close supervisory relationships in shaping undergraduate research outcomes, participants emphasized individualized support as critical to skill development and confidence [22, 23, 24]. The preference for a hybrid model of engagement reflects broader shifts in educational delivery following the COVID‐19 pandemic, where flexibility and accessibility have become increasingly important for equity and participation [25]. However, these preferences should be interpreted cautiously, as they may partly reflect cohort‐specific experiences rather than enduring pedagogical norms.

From an implementation perspective, the sustained delivery of the programme over multiple years, coupled with consistent scholarly outputs, suggests feasibility within routine academic structures. Framing the evaluation using implementation science concepts allows this study to move beyond satisfaction metrics and contributes to understanding how undergraduate research initiatives can be embedded within clinical departments rather than operating as isolated or opportunistic electives.

### 4.1 Methodological Considerations and Limitations

One of the strengths of this study is its contribution to the relatively underresearched area of student engagement in GP research. The inclusion of both quantitative and qualitative data provides a comprehensive view of the programme's effectiveness. The use of the Kirkpatrick evaluation model helped structure the survey to assess both the feasibility and acceptability of the programme from the participants' perspective.

However, there are some limitations. First, there could be a response bias where the participants with stronger interest in primary care research may have been more likely to respond to the survey. Furthermore, the majority of the survey responses came from students who were still in their medical studies, which may not fully represent the experiences of those who have completed the programme and entered clinical practice. The small sample size limits the generalizability of the findings, and qualitative data from open‐ended surveys may have limited analytical depth. Full psychometric validation of the instrument was not feasible. Future studies with larger cohorts and a more diverse range of participants could offer more robust insights.

### 4.2 Implications for Practice and Policy

This study has implications not only for the UCD GP SSRA programme but also for the wider design of undergraduate research training within clinical education. Participants' emphasis on individualized supervision, flexible delivery and the availability of both in‐person and remote engagement highlights a model that is adaptable across diverse institutional and geographic contexts. Hybrid research training structures combining one‐to‐one mentorship, small‐group learning and online participation offer a scalable approach that can support equity, reduce barriers to participation and accommodate students who are geographically distant, time‐constrained or from underrepresented backgrounds.

The findings also underscore the value of GP as a research‐active learning environment [26]. Unlike hospital‐based or laboratory‐focused research programmes, GP‐based research exposes students to population health, continuity of care, health systems and patient‐centred inquiry. These features align closely with contemporary priorities in medical education and health policy, where integrated, community‐based and preventive care models are increasingly emphasized. Embedding research within GP therefore allows students to develop methodological and analytical skills while simultaneously engaging with real‐world clinical complexity, making this model particularly transferable to other primary care and community‐based training settings.

Whereas the current evaluation focused on medical students within a GP setting, the structural components of the programme, one‐to‐one mentorship, hybrid delivery and community‐embedded inquiry provide a scalable blueprint for other health disciplines. In an increasingly integrated healthcare landscape, the ability to conduct research within primary care is a core competency not only for physicians but also for nursing, pharmacy and allied health professionals [27]. By adopting this model, other clinical departments can move from ‘isolated’ research electives towards an integrated ‘practitioner‐scholar’ approach [28]. Future iterations could involve ‘paired’ research projects where, for example, a medical student and a pharmacy student collaborate on a medication adherence study within a primary care setting [29]. Such a model would not only enhance research capability but also build the collaborative competencies required for modern clinical practice.

From a policy perspective, the demonstrated gains in research skills, scholarly confidence and interest in academic and GP career pathways suggest that structured undergraduate research programmes can function as early capacity‐building mechanisms for the primary care research workforce. This is especially important given ongoing international concerns about the sustainability of academic GP and the underrepresentation of primary care in clinical research. Providing protected time, mentorship and funding at undergraduate level may help normalize research engagement as part of future GP practice rather than as an optional or elite activity.

Finally, this study also highlights the importance of rigorous evaluation frameworks and validated measurement tools in assessing educational innovation. The use of established models to structure both programme delivery and outcome assessment provides a foundation for replication and benchmarking across institutions. As future cohorts are evaluated using validated research self‐efficacy instruments, this programme will generate more robust evidence on how GP‐based research experiences influence skills, confidence and career trajectories. Together, these elements support the adoption of this model in broader educational and policy contexts, strengthening the case for primary‐care‐embedded research training as a core component of modern medical education.

## 5 Conclusion

The GP SSRA programme has shown positive outcomes, increasing student engagement in research and reported enhanced interest in both primary care research and GP. More generally, the findings suggest that integrating structured, mentored research opportunities into clinical education can enhance research skills, support evidence‐based practice and enrich career development across specialties. Moving forward, the adoption of such models beyond medicine could foster a culture of interprofessional scholarship, where students from various backgrounds collaborate on population health and patient‐centred inquiry. Prospective–retrospective comparison of research confidence and capability using a validated research self‐efficacy scale such as developed by Bierer et al. [30] can examine changes in students' research self‐efficacy, career motivations and perceptions of GPs; strengthen causal inference; support instrument validation; and provide insight into the longer term educational impact of GP‐based undergraduate research programme.

## Author Contributions

**Nandakumar Ravichandran:** conceptualization, data curation, formal analysis, investigation, methodology, project administration, resources, software, validation, visualization, writing – original draft, writing – review and editing. **Crea Carberry:** data curation, formal analysis, investigation, methodology, supervision, validation, visualization, writing – original draft, writing – review and editing. **Nia Clendennen:** validation, writing – original draft, writing – review and editing. **John Broughan:** project administration, validation, writing – original draft, writing – review and editing. **Geoff McCombe:** project administration, validation, writing – original draft, writing – review and editing. **Walter Cullen:** conceptualization, data curation, funding acquisition, investigation, methodology, project administration, resources, software, supervision, validation, visualization, writing – original draft, Writing – review and editing.

## Funding

The authors have nothing to report.

## Conflicts of Interest

The authors declare no conflicts of interest.

## Data Availability

The data associated with this study are available in the Appendix A. The open and closed responses for this study cannot be shared in the future under any circumstance with persons not named on approved ethical review documentation (including being shared publicly in a data repository). While deidentified to the greatest possible extent, the study data contain details (e.g., accounts of unique characteristics and experiences) that would have a high risk of breaching the participants’ privacy and confidentiality. Anonymous and aggregated details regarding the numbers of participants that took part in the study and their basic characteristics are presented within the manuscript as these details do not compromise privacy and confidentiality and were agreed to by participants that signed study consent forms.

## Appendix A:

### Study Instrument (Self‐Administered Questionnaire)

1 Age
2 Gender
3 What specialty are you currently practising in?
4 I was satisfied with this SSRA programme in General Practice.

*Strongly agree* to *strongly disagree*

5 My SSRA project was interesting

*Strongly agree* to *strongly disagree*:

6 My research skills improved as a result of taking part in this SSRA programme.

*Strongly agree* to *strongly disagree*:

7 This SSRA programme increased my motivation to pursue a career in research.

*Strongly agree* to *strongly disagree*:

8 This SSRA programme increased my motivation to pursue a career in general practice.

*Strongly agree* to *strongly disagree*:

9 I expanded my professional network by taking part in this SSRA programme.

*Strongly agree* to *strongly disagree*:

10 My supervisors on the SSRA programme were helpful.

*Strongly agree* to *strongly disagree*:

11 Which aspects of the SSRA programme did you like the most? (Tick all that apply)

Webinars, group sessions, individual meetings with supervisors:

12 Which aspects of the SSRA programme did you like the least? (Tick all that apply)

Webinars, group sessions, individual meetings with supervisors, none:

13 I liked the fact that I could participate in this SSRA programme remotely (i.e., via Zoom and email)

*Strongly agree* to *strongly disagree*:

14 What was good about the SSRA programme in general practice?
15 How has this experience influenced your overall attitude towards research?
16 How has this experience influenced your overall attitude towards working in general practice?
17 What specific skills or competencies do you feel you've developed through this experience, and how might these be valuable in general practice?
18 How could it be improved?
19 Do you have any other feedback on this programme that you would like to mention?
